# Co-infection paludisme et COVID-19 chez les patients admis au service d’infectiologie COVID du Centre Hospitalier Universitaire de Libreville

**DOI:** 10.11604/pamj.2022.41.101.28751

**Published:** 2022-02-04

**Authors:** Marielle Igala, Elsa Ayo Bivigou, Ulrich Davy Kombila, Stéphanie Ntsame Ngoua, Jean Felix Ngomas, Adrien Mougougou, Arsène Ifoudji Makao, Charlene Manomba, Irène Augustine Mistoul, Gabrielle Atsame Ebang, Anita Akiko Mbourou, Metogho Essandone, Liliane Flore Pemba, Annick Flore Mfoumou, Fifi Claire Ada Loembe, Léonard Kouegnigan Rerambiah, Jean Bruno Boguikouma, Marielle Karine Bouyou Akotet

**Affiliations:** 1 Service d’Infectiologie COVID, Centre Hospitalier Universitaire de Libreville, Libreville, Gabon,; 2 Laboratoire d´Hématologie et de Biochimie, Centre Hospitalier Universitaire de Libreville, Libreville, Gabon,; 3 Service de Radiologie, Centre Hospitalier Universitaire de Libreville, Libreville, Gabon,; 4 Service de Gynéco-obstétrique, Centre Hospitalier Universitaire de Libreville, Libreville, Gabon,; 5 Service de Pédiatrie, Centre Hospitalier Universitaire de Libreville, Libreville, Gabon,; 6 Département de Parasitologie-Mycologie, Université des Sciences de la Santé, Libreville, Gabon

**Keywords:** Co-infection, paludisme, COVID 19, Co-infection, malaria, COVID-19

## Abstract

Le travail visait à rapporter les cas de co-infection paludisme - COVID-19, après la recherche systématique de plasmodium chez les patients pris en charge dans les services d´infectiologie COVID (SiCOV) du Centre Hospitalier Universitaire de Libreville (CHUL). Il s´est agi d´une étude prospective, observationnelle menée d´Avril à Juillet 2020 dans les SiCov du CHUL. Les patients des deux sexes, âgés de plus de 18 ans avec un test de réaction de polymérisation en chaîne (PCR) positif pour le SARS-CoV-2 dont le résultat de la goutte épaisse était disponible, ont été inclus. Pour chaque patient les données démographiques (âge, sexe, poids, taille), les antécédents et les examens clinique et biologique étaient reportées dans le fichier excel. Sur un total de 253 patients qui répondaient aux critères d´inclusions, 8 faisaient un accès palustre associé à une PCR à SARS-CoV-2 positive. Il s´agissait de 3 femmes et de 5 hommes d´un âge moyen de 36,9 ans (25-53 ans). Le mode de contamination était inconnu pour 7/8. Tous les patients étaient fébriles, 6/8 avaient des céphalées et 5/8 présentaient une gêne respiratoire. Moins de la moitié des patients avaient des manifestations oto-rhino-laryngées (anosmie, ageusie) ou digestives (diarrhée). Le seul patient qui présentait une forme sévère est décédé au 5^e^ jour de l´hospitalisation. Les similitudes cliniques entre le paludisme et la COVID-19 peuvent prêter à confusion en zone d´endémie palustre. La co-infection paludisme- COVID-19 n´a pas donné lieu à des formes cliniques graves.

## Abstract (en)

The purpose of this study was to report the cases of co-infection of malaria and COVID-19, after systematic search for plasmodium in patients treated in the COVID Infectious Disease Department (SiCOV) of the Libreville University Hospital (LUH). We conducted a prospective, observational study in the LUH SiCOV from April to July 2020. Patients of both sexes, aged over 18 years, with positive Polymerase Chain Reaction (PCR) test for SARS-CoV-2 with thick blood smear result available, were included. For each patient, demographics (age, gender, weight, height), history and clinical and biological examination results were reported in the Excel file. Of a total of 253 patients who met the inclusion criteria, 8 had malaria associated with positive SARS-CoV-2 PCR. These were women (3) and men (5), with an average age of 36.9 years (25- 53 years). The mode of transmission was unknown in 7/8. All patients were febrile, 6/8 had headaches and 5/8 had respiratory discomfort. Less than half of patients had otolaryngeal (anosmia, ageusia) or digestive (diarrhea) manifestations. One patient with severe form died on day 5 of hospitalization. Clinical similarities between malaria and COVID-19 can lead to confusion in malaria endemic areas. The co-infection of malaria and COVID-19 did not result in severe clinical forms.

## Introduction

Le SARS-CoV-2, initialement apparu dans la ville de Wuhan en Chine en décembre 2019, s´est répandu à travers le monde pour donner lieu à une pandémie mondiale. L´Organisation Mondiale de la Santé (OMS) a rapporté en juillet 2020, 16 114 449 cas avec un nombre de décès estimé à 646 641 [1]. Dans la même période en Afrique le virus a été responsable de 712 920 cas d´infection et 11 900 décès. La principale hypothèse physiopathologique de l´infection est celle de la réaction inflammatoire générée par le virus. En effet, la réponse inflammatoire sévère appelée « tempête de cytokines » se caractérise par une augmentation du niveau des interleukines et du facteur de nécrose tumorale alpha responsable d´un syndrome de détresse respiratoire aiguë [2]. Lorsque le SARS-CoV-2 infecte un individu, il ne se limite pas aux poumons, mais s´attaque aussi aux autres organes en se liant à des récepteurs cellulaires présents à la surface des tissus [3]. Les formes sévères et graves de la maladie concernent les sujets âgés et ceux porteurs de comorbidités telles que l´hypertension artérielle, le diabète, les maladies cardiovasculaires et respiratoires [2]. Les principales manifestations cliniques de la maladie sont la fièvre, la toux, la gêne respiratoire, les douleurs pharyngée et articulaire, l´asthénie, la diarrhée et d´autres symptômes [4, 5].

Comme pour toute infection virale, un risque de co-infection ou de surinfection est à craindre. Ainsi les bactéries responsables d´infections respiratoires peuvent s´associées au SARS-CoV-2 et seraient responsables de décès des patients admis en unité de soins intensifs [6, 7]. Des co-infections virales et fongiques ont également été rapportées. Très peu de cas d´association avec un parasite sont publiés [8]. La propagation de la COVID-19 à travers le monde a fait craindre à l´OMS un scénario catastrophe pour l´Afrique et une perte d´intérêt pour des maladies aussi meurtrières que la COVID-19 telle que le paludisme [9]. Pour l´année 2018 l´OMS a estimé à 228 millions le nombre de cas de paludisme dans le monde avec près de 405 000 décès, dont plus de 90% en Afrique subsaharienne [1]. Au Gabon la transmission du paludisme est pérenne, la prévalence d´environ 36% à Libreville augmente chez les grands enfants et les adultes [10, 11]. Le caractère endémique de l´infection dans le pays conduit à la recherche systématique d´une infection à *plasmodium falciparum* devant toute fièvre. C´est conscient de la nécessité d´exclure un paludisme et dans le souci de conserver une démarche diagnostique cohérente que de nombreux grands centres hospitaliers de la capitale ont inclus la réalisation d´un test de diagnostic rapide (TDR) et/ou une goutte épaisse dans le panel d´examens pour tous les patients suspects d´infection à SARS-CoV-2 dès le début de l´épidémie. La présente étude avait pour but de rechercher le degré de gravité de l´association paludisme - infection à COVID-19 à travers 8 observations de patients pris en charge dans les services d´infectiologie COVID (SiCov) du Centre Hospitalier Universitaire de Libreville (CHUL).

## Méthodes

Il s´est agi d´une étude transversale, menée d´avril à juillet 2020 dans les SiCov du CHUL. Les patients des deux sexes, âgés de plus de 18 ans admis aux SiCov avec un test PCR positif pour le SARS-CoV-2 et dont le résultat de la goutte épaisse était disponible, ont été inclus. Les patients dont la goutte épaisse n´avait pas été réalisée n´ont pas été inclus. Pour chaque patient les données démographiques (âge, sexe) et anthropométriques (poids et taille), les antécédents de diabète, d´hypertension artérielle (HTA) et les données de l´examen clinique étaient reportés dans une fiche de recueil destinée à cet effet. Le mode de contamination était recherché. Il pouvait être connu (sujet contact d´une personne infectée ou un professionnel de santé) ou inconnu. L´examen clinique à l´admission reprenait les principaux symptômes, leur délai d´apparition, une prise de température, de tension artérielle et de la saturation en oxygène. Il était complété par un examen physique détaillé de l´organe responsable du principal point d´appel. Au terme de l´examen l´état clinique des patients était classé en: forme pauci-symptomatique; forme modérée: signes de pneumonie faite de fièvre, toux, dyspnée et polypnée avec une saturation de l´oxygène à l´air ambiant au-delà de 90%; forme sévère: signes cliniques de pneumonie (fièvre, toux, dyspnée, respiration rapide) plus l´un des signes ou symptômes suivants: fréquence respiratoire > 30 respirations/min; détresse respiratoire sévère; ou SpO_2_ < 90% en air ambiant; forme critique: apparition dans la semaine suivant un accident clinique connu (à savoir, une pneumonie) ou la survenue ou l´aggravation de symptômes respiratoires. Apparition d´un dysfonctionnement aigu d´un organe, engageant le pronostic vital, dû au dérèglement de la réaction de l´hôte à une infection présumée ou avérée.

Les examens complémentaires comprenaient des prélèvements naso et oro-pharyngés pour le diagnostic de l´infection à SARS-CoV-2 à partir des kits Real Time Fluorescent RT- PCR Labs for detecting SASR-Cov-2, une goutte épaisse qui permettait de determiner le type de plasmodium concerné, un hémogramme, un bilan d´hémostase, le dosage quantitatif de la CRP, un bilan hépatique complet, un bilan rénal, un ionogramme, un électrocardiogramme et une tomodensitométrie thoracique. En cas de goutte épaisse positive, le traitement anti-palustre par les sels de quinine ou l´association arthémeter/luméfantrine était initié avant celui de l´infection à SARS-CoV-2 fait de zinc, vitamine C, une prévention anti-thrombotique et azythromycine sans hydroxychloroquine (dispensé à l´ensemble des patients sauf en cas d´accès palustre associé). L´ensemble des données de l´étude ont été rapportées sur le fichier Excel 2013.

## Résultats

Un total de 253 patients répondait aux critères d´inclusion durant la période de l´étude parmis lesquels 8 (3.16%) avaient une goutte épaisse positive à *plasmodium falciparum*. Les caractéristiques générales et biologiques des patients sont consignées dans les Tableau 1 et Tableau 2. Il s´agissait de 3 femmes et de 5 hommes d´un âge moyen de 36,9 ans (25-53 ans). Le mode de contamination était inconnu pour 7/8. Le diabète et l´hypertension artérielle étaient les comorbidités retrouvées chez trois patients. Une des 3 femmes était au troisième trimestre de grossesse. Le tableau clinique était dominé par la forme modérée chez 7 des 8 patients. Tous les patients étaient fébriles, 6/8 avaient des céphalées et 5/8 présentaient une gêne respiratoire. Moins de la moitié des patients avaient des manifestations oto-rhino-laryngées (anosmie, ageusie) ou digestives (diarrhée). Sur le plan biologique l´anémie était modéré pour deux patients (11,7 et 9,5 g/dl) et sévère pour un (6,7 g/dl). La thrombopénie était retrouvée dans 4/8 cas dont 3 à moins de 100 000/mm^3^. La protéine C-réactive (CRP) était élevée chez tous les patients chez qui elle avait été dosée. Un traitement anti-palustre à base d´artemether/luméfantrine a été initié chez 6 des 8 patients et des sels de quinine chez 2 patients. Il était immédiatement suivi par celui du SARS-CoV-2 fait de l´association vitamine C, Zinc et Clarythromycine (Tableau 3). Après une durée moyenne de 12,6 jours (5-17 jours), les patients dont l´évolution clinique avait été jugée favorable ont été autorisés à regagner leur domicile. Le seul patient qui présentait une forme sévère est décédé au 5^e^ jour de l´hospitalisation (Tableau 3).

**Tableau 1:** caractéristiques générales des patients infectés

	Cas 1	Cas 2	Cas 3	Cas 4	Cas 5	Cas 6	Cas 7	Cas 8
Age en années	30	41	49	35	29	25	53	33
Sexe masculin	F	M	M	M	F	F	M	M
**Mode de contamination**								
Cas confirmé					Oui			
Professionnel de santé								
Cas contact								
Inconnu	Oui	Oui	Oui	Oui		Oui	Oui	Oui
**Comorbidités**								
HTA				Oui		Oui		Oui
Diabète			Oui				Oui	
IMC >30		Oui				Oui		
Insuffisance rénale								
Grossesse	Oui (15SA)*							
Tabagisme								
**Symptômes à l´admission**								
Toux	Oui	Oui	Oui	Oui		Oui	Oui	
Dyspnée	Oui	Oui	Oui	Oui			Oui	
Fièvre	Oui	Oui	Oui	Oui	Oui	Oui	Oui	Oui
Agueusie							Oui	
Anosmie			Oui	Oui			Oui	
Céphalées	Oui		Oui	Oui	Oui	Oui	Oui	
Diarrhée		Oui						
Douleurs		Oui				Oui		
articulaires/thoraciques	Oui	Oui	Oui			Oui		
**Forme clinique**	FM**	FM	FM	FM	FM	FM	FM	FC**

*SA: semaine d´aménorrhée **FM: forme modérée FC: forme critique

**Tableau 2:** caractéristiques biologiques des patients infectés

	Cas 1	Cas 2	Cas 3	Cas 4	Cas 5	Cas 6	Cas 7	Cas 8
Leucocytes (G/L)	5	3.9	5	8	4.7	4.73	10	12.4
Neutrophiles (G/L)	3.43	1.8	3.582	5.84	3.8	3.12	7.2	6.163
Lymphocytes (G/L)	1.28	1.4	0.95	1.76	0.7	0.71	2.3	4.464
RNL	2.7	1.3	3.8	3.3	5.4	4.4	3.1	1.3
Monocytes (G/L)	0.28	0.6	0.29	0.4	0.2	0.52	0.4	1.423
Eosinophiles (G/L)	0.01	0.1	0.1	0	0	0.37	0.1	0.3
Basophiles (G/L)	0	0	0.078	0	0	0.01	0	10
Hémoglobine (g/dl)	12	13,9	13,9	13	12 ,1	9,5	11,7	6,7
Plaquettes (G/L)	93	144	180	207	85	87	161	164
CRP (mg/l)	21,2	13,7	NR	NR	388,2	36,2	48	NR
Parasitémie® (p/ml)	87	150	55	100	110	78	95	65

NR°: non réalisé ® Tous les patients étaient infectés par plasmodium falciparum

**Tableau 3:** traitement et devenir des patients

	Cas 1	Cas 2	Cas 3	Cas 4	Cas 5	Cas 6	Cas 7	Cas 8
Traitement du paludisme Art/lum* Quinine IV	oui	oui	oui	oui	oui	oui	oui	oui
Traitement du SARS-CoV-2	oui	oui	oui	oui	oui	oui	oui	oui
Durée d´hospitalisation	17	10	13	13	19	12	10	5
Complication	Non	Non	Non	Non	Non	Non	Non	oui
Devenir du patient	Guérison	Guérison	Guérison	Guérison	Guérison	Guérison	Guérison	Décès

Art/lum* : Artemether/ luméfantrine

## Discussion

Ce travail est l´un des rares portants sur la co-infection paludisme et COVID-19 en zone d´endémie palustre. Les données générales des 8 patients co-infectés, retrouvaient une population jeune avec des co-morbidités dont certaines les rendaient vulnérables au paludisme et/ou à la COVID-19. La première d´entre elle était la grossesse. Une patiente porteuse d´une grossesse au premier trimestre a présenté une forme modérée de l´infection à COVID-19. L´association des deux infections n´a pas donné lieu à une présentation clinique grave même s´il est admis que le paludisme est responsable d´une morbi-mortalité au cours de la grossesse. Au Gabon l´incidence des formes graves est en diminution depuis l´implémentation des mesures préventives tant médicamenteuse que clinique [12, 13]. La gravité de l´infection à SARS-CoV-2 au cours de la grossesse est très diverses. Il y a autant de cas graves que modérés quel que soit le terme de la grossesse [14-16]. La fièvre était le symptôme présenté par l´ensemble des patients. Elle est connue pour être une des principales manifestations du paludisme de l´adulte au Gabon. Bouyou Akotet *et al*. sur 304 patients de plus de 15 ans vus en consultation pour une fièvre, retrouvaient une infection à plasmodium dans 42,1% des cas [10]. À la connaissance de ces résultats, on aurait pu s´attendre à un nombre plus important que les seuls 3.16% de goutte épaisse positive.

D´autres manifestations communes au paludisme et à la COVID-19 existaient: céphalées, douleurs articulaires, diarrhée. En effet, les premières descriptions cliniques de l´infection à SARS-CoV-2 désignaient la fièvre, la toux, la perte de l´odorat et du goût, les céphalées, les myalgies, les douleurs articulaires et la diarrhée [4]. Certains de ces symptômes peuvent être observés au cours du paludisme en cas de co-infection avec un virus ou une bactérie. C´est notamment le cas de la toux et des manifestations oto-rhini-laryngés. La similitude des tableaux cliniques entre les deux affections n´a pas permis d´emblée de les distinguer. Si le paludisme a souvent été évoqué, une autre infection qui lui était associée a été soupçonnée, et dans le cas présent de pandémie, il s´agissait en premier de la COVID-19. La quasi-totalité des patients a présenté une forme modérée de la maladie alors que le pire était attendu. En effet, la physiopathologie des deux affections fait état d´une réaction inflammatoire majeur : activation du système immunitaire et avec lui les cytokines pro-inflammatoires, le TNFÎ±, l´interferon-gamma et les interleukines pour le paludisme et un « orage cytokinique » pour la COVID-19 [2]. Cette relative modération des formes cliniques pourrait venir du jeune âge des patients compris entre 23 et 53 ans qui les excluaient d´emblée des formes graves du paludisme et l´effet immuno-modulateur et protecteur du plasmodium face à certains virus respiratoires [2]. Le recours aux examens complémentaires a été indispensable pour faire la preuve de l´une ou l´autre affection. Les patients ont ainsi été testés à la fois pour le paludisme et le SARS-CoV-2. Le diagnostic de paludisme ne faisait aucun doute grâce à l´utilisation de méthodes qui ont fait la preuve de leur efficacité (TDR et goutte épaisse) et dont la pratique est bien répandu tant à Libreville que dans les zones rurales [17].

L´analyse de l´hémogramme retrouvait aussi bien des anomalies fréquentes au cours du paludisme (anémie et thrombopénie) que celles dont l´interprétation aide au diagnostic de sévérité de l´infection à SARS-CoV-2. C´est ainsi qu´une leucocytose a été observée chez un patient qui présentait la forme clinique la plus sévère de la maladie. Le nombre de leucocytes et de neutrophiles sont de bons indicateurs de la gravité de la COVID et de l´existence d´une surinfection bactérienne [18]. Dans le cas précis de ce patient la surinfection bactérienne ne pourrait être formellement exclue, car ni le paludisme ni le SARS-CoV-2 ne pouvait expliquer l´hyperleucocytose. Le traitement du SARS-CoV-2 est un sujet à controverse. Au début de la pandémie de nombreux pays ont fait le choix de l´utilisation de la chloroquine ou de l´hydroxychloroquine en raison de leur action antivirale [19, 20]. Au Gabon, les patients pris en charge dans les différents SICOV à travers le pays ont été traités selon le protocole associant chloroquine ou hydroxychloroquine-azithromycine-zinc et vitamine C. L´existence d´une co-infection au paludisme chez certains patients a conduit à une reconsidération du traitement. En effet l´apparition de résistances à la chloroquine a entrainé il y a plusieurs années l´arrêt de son utilisation au cours des infections palustres au profit des dérivés de l´artésunate et la quinine [21]. L´évolution de la co-infection a été simple pour l´ensemble des patients qui présentaient une forme modérée du SARS-CoV-2. Elle n´a pas donné lieu à des complications. Le caractère modéré des présentations cliniques a conduit à des hospitalisations d´une durée d´environ 10 jours. Pour un patient, l´hospitalisation a été raccourcie par l´existence d´une insuffisance rénale rapidement fatale. L´imputabilité de cette complication au SARS-CoV-2 ou au paludisme reste difficile à déterminer, car chacune des infections peut en être responsable. Elle entre dans la définition des formes sévères dans les deux cas [22].

## Conclusion

Les similitudes cliniques entre le paludisme et la COVID-19 peuvent prêter à confusion en zone d´endémie palustre. La réalisation systématique d´un TDR ou d´une goutte épaisse doit demeurer en pratique courante devant toute fièvre même en période d´épidémie. La co-infection paludisme - COVID-19 n´a pas donné lieu à des formes graves. Le rôle répresseur du paludisme lors de son association avec une infection virale pourrait être une des hypothèses.

### Etat des connaissances sur le sujet

- Alors que l´infection à SARS-CoV-2 ne cesse de faire des morts à travers le monde et que les connaissances sur le mode d´action du virus ont évolué, très peu de travaux se sont intéressés à son association avec certains parasites dont le plus mortel est le paludisme.

### Contribution de notre étude à la connaissance

- Dans les pays à forte prévalence du paludisme la morbi-mortalité liée au parasite n´a pas disparue durant la pandémie mondiale due au SARS-CoV-2;
- La connaissance du profil clinique et biologique de la co-infection des deux germes devrait amener de nombreux pays à maintenir sinon à renforcer leurs stratégies diagnostiques.

## Remerciements

Tout le personnel du CHUL qui a activement pris part à la gestion des patients infectés par le SARS-CoV-2.
